# In Memoriam: James Harlan Steele (1913–2013)

**DOI:** 10.3201/eid2003.IM2003

**Published:** 2014-03

**Authors:** Myron G. Schultz

**Affiliations:** Centers for Disease Control and Prevention, Atlanta, Georgia

**Keywords:** in memoriam, Centers for Disease Control and Prevention, James Harlan Steele, James Steele, veterinarian

James Steele, DVM, MPH, passed away on November 10, 2013, in Houston; he was 100 years old. Jim Steele (Figure) was an extraordinary man. All of the dimensions of his life were on a grand scale. He was larger than life in so many ways; his vision, his leadership, his accomplishments in public health, his worldwide friendships, his mentorship of scores of young acolytes who came within his orbit, his extraordinary memory, his bear hugs, and his longevity were all manifestations of his boundless enthusiasm for life. 

**Figure:**
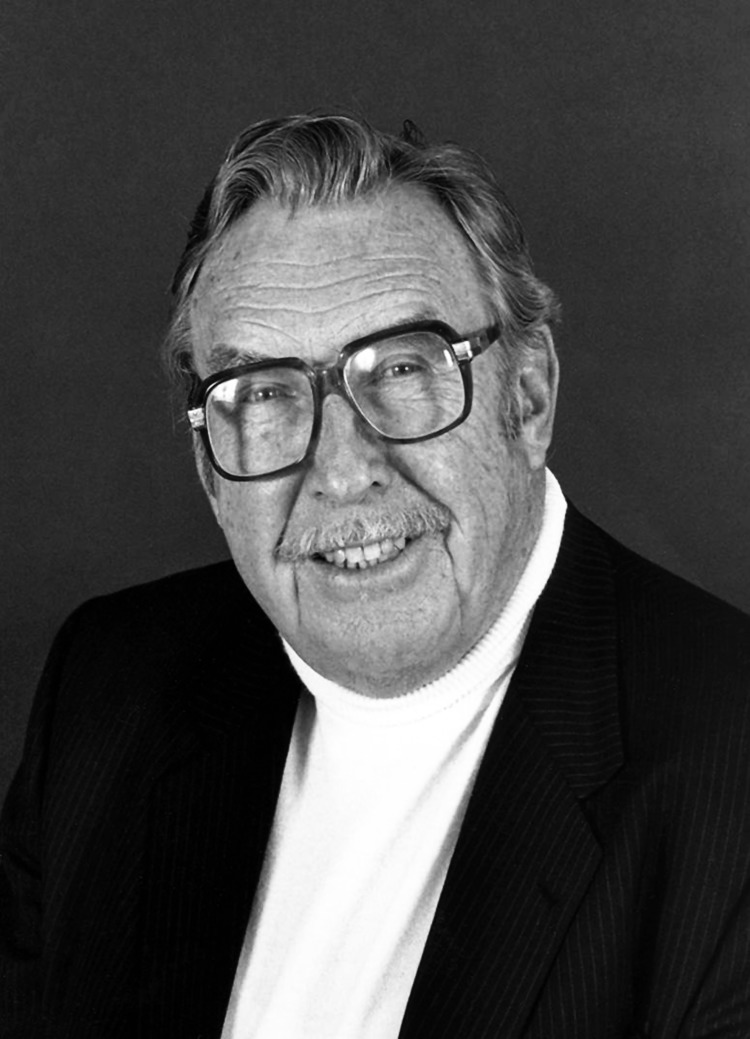
James Harlan Steele.

Dr Steele’s professional career spanned more than 70 years. It began in 1938 when he worked in a brucellosis testing laboratory for the Michigan State Department of Agriculture while studying veterinary medicine at Michigan State University. Brucellosis developed in many of his veterinary colleagues, and he wanted to learn how the causative pathogen and other pathogens were transmitted from animals to humans. This was the beginning of his lifelong vocation of studying and controlling zoonotic diseases.

In 1941, Dr Steele received a doctorate of veterinary medicine from Michigan State University, and in 1942, he earned a master of public health degree from Harvard University. In 1943, he was commissioned as a sanitarian in the Public Health Service (PHS). He spent most of World War II in Puerto Rico and the Virgin Islands, where he coordinated milk and food sanitation programs, evaluated zoonotic threats to the islands, and conducted research on brucellosis, bovine tuberculosis, rabies, and Venezuelan equine encephalitis.

After the war, Dr Steele’s encounter with Assistant Surgeon General Joseph Mountin, the legendary founder of the Communicable Disease Center (now named Centers for Disease Control and Prevention; CDC), changed his career. Dr Steele was fond of telling how Dr Mountin challenged him by asking, “What are you veterinarians going to do now that the war is over?” In response, Dr Steele described some of the known zoonotic diseases, and Dr Mountin asked questions about their prevalence and control. Dr Steele’s main response was “We don’t have any data, nor do we know how to control these zoonoses*.*” In the end, Dr Mountin said, “Steele, it is quite apparent that we have a problem and a lot of ignorance—let us exploit it!” Thus, in 1945, Dr Steele produced a detailed report titled Veterinary Public Health, which outlined the risks posed by zoonotic diseases and the benefits of employing veterinarians for research and response efforts. Dr Mountin and Surgeon General Thomas Parran were impressed by the scope of the report, and in 1947, Dr Steele convinced the Surgeon General to establish a Veterinary Medical Officer category in the PHS. He entered this service category and became the PHS chief veterinary officer. When he retired from the PHS Commissioned Corps in 1971, Dr Steele was an Assistant Surgeon General, the first veterinarian to achieve this rank.

 Dr Steele came to CDC in 1947, just after its beginning. There was no road map for the work he did—he was a pioneer, creating CDC’s veterinary public health program. Much of his work was focused on rabies eradication. He and his team improved the existing vaccine, and he then worked toward eliminating the disease in dogs and cats in the United States and other countries. He also worked on other diseases that threatened humans and animals, including bovine tuberculosis and brucellosis, Q fever, psittacosis, salmonellosis and other food-borne diseases, and avian influenza.

Dr Steele also pioneered the integration of veterinary public health into the Pan American Health Organization (PAHO) and the World Health Organization (WHO). In 1950, he attended the first WHO Expert Committee meeting, and in 1965, he chaired the second meeting. These meetings brought together the most eminent experts in the world of zoonotic diseases and emphasized the need for international collaboration and common goals. Jim Steele worked closely with PAHO and WHO throughout his career.

Dr Steele enjoyed a good relationship with Dr Alexander Langmuir, who founded CDC’s Epidemic Intelligence Service (EIS) training program in 1951. In 1953, Dr Langmuir asked Dr Steele to recruit veterinarians to work in all epidemiologic areas (animal and nonanimal diseases) of the EIS program. This was the beginning of a new sphere of opportunity for veterinarians in public health. Today, veterinarians are integrated into all areas of PHS activity.

 When he retired from PHS in 1971, Dr Steele became a professor at the University of Texas School of Public Health. He was an active teacher, writer, and mentor. He compiled the CRC Handbook Series in Zoonoses, the first comprehensive collection addressing diseases shared by humans and animals. The book remains a staple of public health curricula throughout the world. 

Dr Steele received numerous awards during his career. Among them are the American Public Health Association's Bronfman Prize, the American Veterinary Medical Association’s International Veterinary Congress Prize, the Surgeon General’s Medallion, the PAHO Abraham Horwitz Award for Excellence in Leadership in Inter-American Health, the OIE (World Organization for Animal Health) Medal of Merit, and many more. In addition, the University of Texas School of Public Health holds an annual James Steele Lecture, and a James H. Steele Veterinary Public Health Award is given annually at CDC’s EIS Conference.

The message of the One Health Initiative is that human health and animal health are inextricably linked: we cannot have good public health unless we have good animal health, and we cannot have good animal health unless we have good public health. Jim Steele was a father of the One Health Initiative. He didn’t merely profess this concept—he practiced it for 7 decades, and he taught it to younger generations of veterinarians. Jim Steele had an extraordinary capacity for mentoring younger health professionals and sustaining lifelong relationships.

Lewis Thomas, the physician–philosopher who wrote about so many aspects of life, said that the highest state of life is to be useful—to be engaged in purposeful activity with your fellow men. By this measure, Jim Steele was a rich man—not in material wealth, which is ephemeral—but in his relationships with other human beings, which are enduring. Jim Steele has left a legacy in which millions of persons have been granted healthier lives. The world is a better place because Jim Steele lived and served humanity.

